# Histological and Pathological Assessment of miR-204 and SOX4 Levels in Gastric Cancer Patients

**DOI:** 10.1155/2017/6894675

**Published:** 2017-01-04

**Authors:** Xiao Yuan, Shuanhu Wang, Mulin Liu, Zhen Lu, Yanqing Zhan, Wenbin Wang, A-Man Xu

**Affiliations:** ^1^Department of General Surgery, The Fourth Affiliated Hospital of Anhui Medical University, Hefei 230022, China; ^2^Department of Gastrointestinal Surgery, The First Affiliated Hospital of Bengbu Medical College, Bengbu 233004, China

## Abstract

Gastric cancer is one of the most common cancers and the efficient therapeutic methods are limited. Further study of the exact molecular mechanism of gastric cancer to develop novel targeted therapies is necessary and urgent. We herein systematically examined that miR-204 suppressed both proliferation and metastasis of gastric cancer AGS cells. miR-204 directly targeted SOX4. In clinical tissue research, we determined that miR-204 was expressed much lower and SOX4 expressed much higher in gastric cancer tissues compared with normal gastric tissues. Associated analysis with clinicopathological parameters in gastric cancer patients showed miR-204 was associated with no lymph node metastasis and early tumor stages whereas SOX4 was associated with lymph node metastasis and advanced tumor stages. In addition, miR-204 and SOX4 were negatively correlated in tissues from gastric cancer patients. Our findings examined the important role of miR-204 and SOX4 played in gastric cancer, and they could be used as candidate therapeutic targets for gastric cancer therapy.

## 1. Introduction

Gastric cancer remains one of the most common cancers and the second leading cause of cancer-related death in the world [1–3]. Most gastric cancers are diagnosed at advanced stages, so efficient therapeutic methods are limited [4]. High recurrence and metastasis rate of gastric cancer is the biggest obstacle [5, 6]. Many studies tried to uncover the mechanism of gastric cancer, but the exact molecular pathways remain unclear. Further study of gastric cancer to develop novel targeted therapies is an urgent issue.

Recently, many studies demonstrated that microRNAs (miRNAs) are responsible for tumorigenesis and metastasis including gastric cancer [7–10]. miRNAs directly interact with the mRNA 3′ untranslated regions (3′UTR) of their target genes including oncogenes and tumor suppressing genes and usually negatively regulate them [11, 12]. In gastric cancer, miR-144, miR-449a, miR-141, miR-361, and so forth were reported to suppress the oncogenicity of tumors [13–16], and miR-19a, miR-223, miR-425, and so forth were demonstrated to promote the oncogenicity of tumors [17–19]. MiR-204 is one of the miRNAs that have been determined to suppress tumor initiation and development in lung cancer, esophageal cancer, gastric cancer, and other cancers [11, 20, 21]. USP47, RAB22A, SIRT1, Snai1, and so forth are targets of miR-204 that mediate miR-204 reducing the oncogenicity of gastric cancer [11, 22, 23]. But there was no fully histological and pathological research about miR-204 in gastric cancer patients in previous studies.

SOX4 belongs to sex-determining region Y (SRY) box family and was recently found to be an oncogene in prostate cancer, colon cancer, lung cancer, bladder cancer, gastric cancer, and so forth [24–29]. SOX4 was also reported to be one target of miR-204 in renal cancer, T-cell acute lymphoblastic leukemia, and* H. pylori*-associated gastric cancer [24, 30, 31]. But correlated expression of miR-204 and SOX4 in gastric cancer tissues from patients remains unknown, and it is necessary for better understanding the functions and mechanisms of miR-204 and SOX4 in gastric cancer patients.

In this study, we systematically examined that miR-204 suppressed both proliferation and metastasis of gastric cancer AGS cells. miR-204 directly targeted SOX4. We collected 54 normal gastric tissues and 54 gastric cancer tissues from patients and evaluated the expression of miR-204 and SOX4, respectively. We found miR-204 was expressed much lower and SOX4 expressed much higher in gastric cancer tissues compared with normal gastric tissues, and miR-204 was associated with no lymph node metastasis and early tumor stages whereas SOX4 was associated with lymph node metastasis and advanced tumor stages. Moreover, miR-204 and SOX4 showed a negative correlation in tissues from gastric cancer patients, which indicated that the pathway miR-204 targeting SOX4 played an important role to suppress tumorigenesis and progression of gastric cancer. Therefore, we provided new evidence that miR-204 and SOX4 can be used as new therapeutic targets for gastric cancer therapy.

## 2. Materials and Methods

### 2.1. Cell Lines and Cell Culture

Human gastric cancer cell AGS was obtained from the American Type Culture Collection (Rockville, MD) and cultured in a humidified incubator at 37°C and 5% CO_2_ as recommended.

### 2.2. Cell Proliferation and Oncogenicity Assays

In this study, we carried out total cell number assay, MTT assay (3-(4,5-dimethylthiazol-2-yl)-2, 5-diphenyltetrazolium bromide), colony formation assay, soft agar colony formation assay, wound healing assay, migration assay, and invasion assay to evaluate cell proliferation and oncogenicity. They were all performed as previously described [32–34].

### 2.3. Luciferase Reporter Assay

Cells were seeded in a 24-well plate and cotransfected with 0.2 *μ*g of psiCHECK2-SOX4 3′UTR or psiCHECK2 control vector and 30 nM miR-204 mimics or its negative control using Lipofectamine 2000. 48 h after transfection, cells were harvested, and reporter assays were performed using a dual luciferase assay system (Promega).

### 2.4. Patients and Tissue Samples

54 normal gastric tissues (nontumorous tissues) and 54 gastric cancer tissues were collected from archive of the Department of Pathology, Anhui Medical University, and the patients underwent surgery at the Second Affiliated Hospital of Anhui Medical University (Hefei, China) between 2013 and 2015. Tumors were graded according to Edmondson-Steiner grading system and staged according to American Joint Committee on cancer staging system [35, 36]. Informed consent documents were got from all of the patients. Research related to patients' tissues was approved by the Institutional Review Board of the Anhui Medical University.

### 2.5. In Situ Hybridization

We performed in situ hybridization in formalin-fixed paraffin-embedded tissues to evaluate the expression of miR-204. Experiments were carried out essentially as previously described [37]. The intensity and the percentage of stained cells were used to score the expression of miR-204 in each section under an Olympus microscope (Olympus America Inc., Melville, NY). 10% or more tumor cells stained were considered as positive expression of miR-204, whereas less than 10% tumor cells stained with any intensity were considered as negative expression.

### 2.6. Immunohistochemistry

Immunohistochemistry analysis in formalin-fixed paraffin-embedded tissues was carried out as described earlier [38]. Rabbit polyclonal antibody against SOX4 (1 : 200, Abcam, Cambridge, UK) was used in this study. The stained sections were scored by two experienced pathologists to calculate the expression of SOX4 under an Olympus microscope. 10% or more tumor cells stained were considered as positive expression of SOX4, whereas less than 10% tumor cells stained with any intensity were considered as negative expression.

### 2.7. Statistical Analysis

In this study, we used SPSS software for Windows (version 13.0; SPSS, Chicago, IL, USA) to perform statistical analyses. The relationship of miR-204 level and SOX4 expression in gastric tissues was analyzed using Pearson's chi-square test. In the statistical analyses, *P* < 0.05 was considered as statistically significant.

## 3. Results

### 3.1. miR-204 Suppressed Proliferation of Gastric Cancer Cells

**Figure 1:**
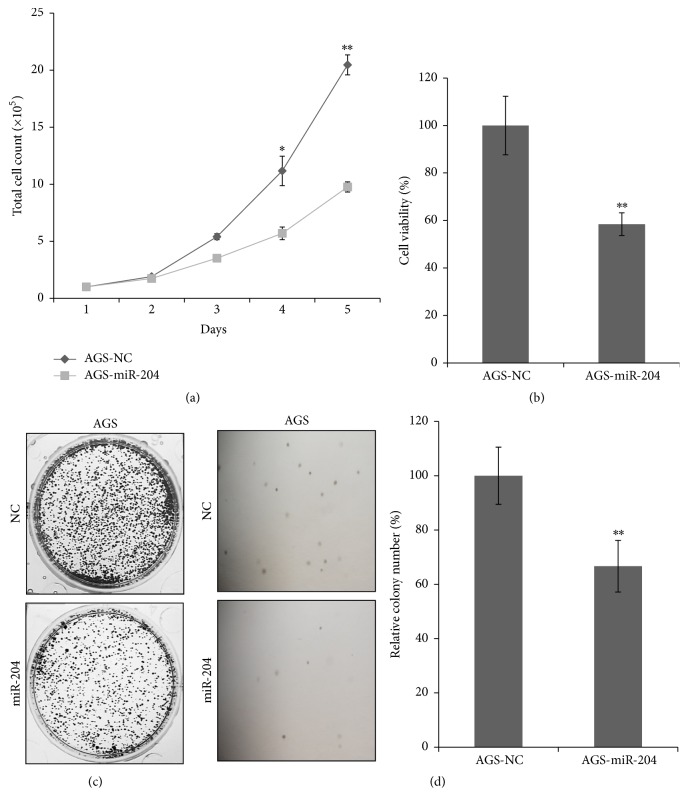
miR-204 suppressed proliferation of AGS cells. AGS cells were transfected with miR-204 or NC mimics to perform cell proliferation assays. (a) Cell total number assay with an original cell number of 100,000; (b) MTT assay; (c) cell colony formation assay; (d) soft agar colony formation assay were performed in AGS cells. ^*∗*^*P* < 0.05. ^*∗∗*^*P* < 0.01.

Recently, several studies demonstrated the important role of miR-204 to suppress tumor initiation and development of several human cancers including gastric cancer [11, 20, 21]. Herein, we systematically examined the role of miR-204 in gastric cancer cells and made an exhaustive analysis of miR-204 in human gastric cancer tissues. In our study, we selected human gastric cancer cell AGS for research. After transfected with miR-204 mimics, total number of AGS cells significantly decreased over a period of 5 days (Figure 1(a)). Concordantly, in MTT assay, cell viability decreased significantly after transfected with miR-204 mimics (Figure 1(b)). In addition, AGS-miR-204 cells showed a decreased cell colony formation compared with AGS-NC cells (Figure 1(c)). Moreover, we carried out soft agar colony formation assay to evaluate the anchorage-independent cell growth ability of AGS cells after transfected with miR-204.  Figure 1(d) showed miR-204 dramatically suppressed the soft agar colony formation. As a whole, miR-204 suppressed proliferation of gastric cancer cells.

### 3.2. miR-204 Suppressed Metastasis of Gastric Cancer Cells

**Figure 2:**
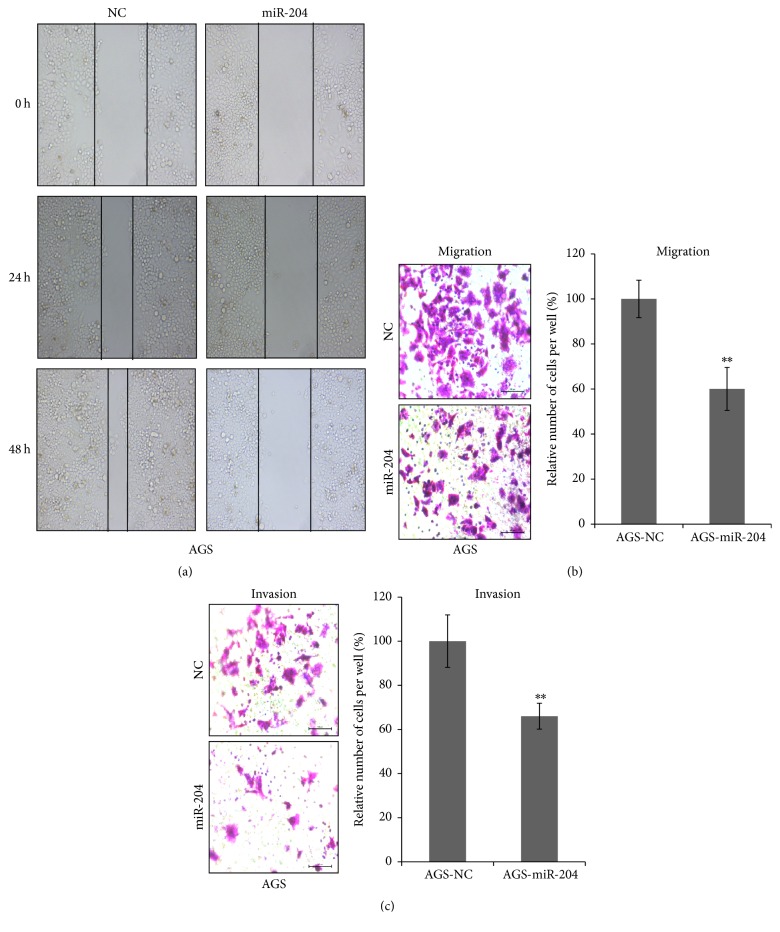
miR-204 suppressed metastasis of AGS cells. (a) Wound healing assay; (b) migration assay; (c) invasion assay were performed in AGS cells after transfected with miR-204 or NC mimics. *P* < 0.05. ^*∗∗*^*P* < 0.01.

We next evaluated the role of miR-204 on metastasis in AGS cells. Firstly, we carried out wound healing assay.  Figure 2(a) showed miR-204 dramatically suppressed the wound closing of AGS cells compared with AGS-NC. Moreover, we performed cell migration and invasion assays in AGS cells. Concordantly, both migration (Figure 2(b)) (AGS-NC 100 ± 8.32% versus AGS-miR-204 60.05 ± 9.52%  *P* < 0.01) and invasion (AGS-NC 100 ± 11.89% versus AGS-miR-204 60.05 ± 5.89%  *P* < 0.01) decreased significantly in AGS cells with forced expression of miR-204 compared with AGS-NC. Therefore, miR-204 also suppressed metastasis of gastric cancer cells.

### 3.3. miR-204 Was Expressed Lower and SOX4 Expressed Higher in Gastric Cancer Tissues Than Normal Gastric Tissues

**Figure 3:**
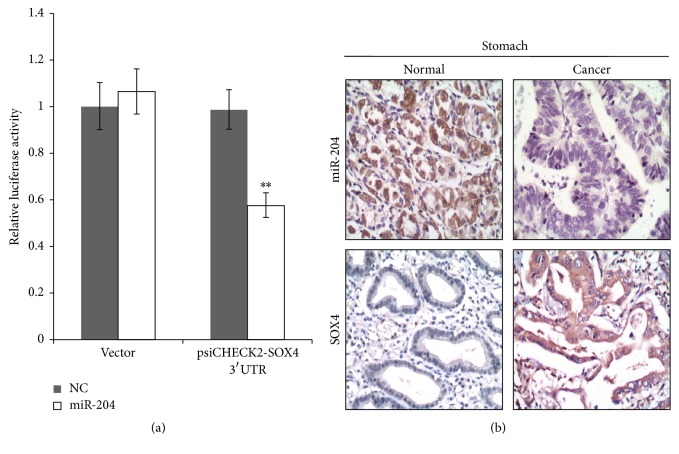
Expression of miR-204 and SOX4 in normal gastric and gastric cancer tissues. (a) Luciferase reporter assay was performed in AGS cells after cotransfected psiCHECK2-SOX4 3′UTR/psiCHECK2 control vector and miR-204 mimics/negative control. (b) Expression of miR-204 in normal gastric and gastric cancer tissues was measured using in situ hybridization. Expression of SOX4 in normal gastric and gastric cancer tissues was measured using immunohistochemistry. Representative pictures showed that miR-204 was expressed much lower in gastric cancer tissues compared with normal gastric tissues, whereas SOX4 was expressed much higher in gastric cancer tissues compared with normal gastric tissues ^*∗∗*^*P* < 0.01.

Since miR-204 suppressed both proliferation and metastasis in gastric cancer cells, we want to know the expression of miR-204 in gastric cancer tissues compared with normal gastric tissues from patients. Zhou et al. and Wu et al. have demonstrated that SOX4 was an important direct target of miR-204 playing an important role to promote the oncogenicity of gastric cancer and renal cancer [24, 30]. We performed luciferase reporter assay to confirm that miR-204 directly targeted the SOX4 3′UTR (Figure 3(a)). In Zhou et al.'s article, they lacked convincible clinical evidence about miR-204 and SOX4 in gastric cancer study. Herein, we collected 54 normal gastric tissues and 54 gastric cancer tissues to analyze the association of miR-204 and SOX4 expression. The expression of miR-204 was measured using in situ hybridization and the expression of SOX4 was measured using immunohistochemistry.  Figure 3(b) showed that miR-204 was expressed much lower in gastric cancer tissues compared with normal gastric tissues, whereas SOX4 was expressed much higher in gastric cancer tissues compared with normal gastric tissues. Moreover, Table 1 showed that, in the 54 gastric cancer tissues, 19 (35.2%) samples were miR-204 positive and 39 (72.2%) samples were SOX4 positive; and in the 54 normal gastric tissues, 34 (63.0%) samples were miR-204 positive and 17 (31.5%) samples were SOX4 positive. These results suggested that patients with gastric cancer preferred a low expression of miR-204 and high expression of SOX4.

### 3.4. Association of miR-204 and SOX4 Expression with Clinicopathological Parameters from Gastric Cancer Patients

For further study, we analyzed the relationship between miR-204 and SOX4 levels and clinicopathological parameters in gastric cancer patients. Clinicopathological parameters in our study involved patients' age, gender, tumor size, lymph node metastasis, tumor grade, and tumor stage. miR-204 level was significantly associated with no lymph node metastasis (*P* = 0.003) and early tumor stages (stages I-II; *P* = 0.048). SOX4 protein level was significantly associated with lymph node metastasis (*P* = 0.001) and advanced tumor stages (stages III-IV; *P* = 0.003). But the associations between expression level of miR-204/SOX4 and patients' age, gender, tumor size, or tumor grade were not significant (Table 2).

### 3.5. Correlation of miR-204 and SOX4 Expression in Gastric Cancer Patients

For further study, we analyzed the correlation of miR-204 and SOX4 expression in gastric tissues from patients. Concordant with former data, miR-204 and SOX4 were statistically negatively correlated in the 54 normal gastric tissues and 54 gastric cancer tissues (*P* < 0.001). Pearson's correlation coefficients were −0.801 and −0.582, respectively (Table 3). Therefore, the pathway miR-204 targeting SOX4 may play a combined role in suppressing gastric cancer development and progression.

## 4. Discussion

In this study, we systematically examined that miR-204 suppressed cell proliferation including cell total number, cell viability, cell colony formation, and soft agar colony formation in gastric cancer cells (Figure 1); miR-204 also suppressed cell metastasis including wound healing, migration, and invasion (Figure 2). miR-204 directly targeted SOX4. We found that miR-204 was expressed much lower in gastric cancer tissues compared with normal gastric tissues by in situ hybridization method, and SOX4 (one target gene of miR-204) was expressed much higher in gastric cancer tissues compared with normal gastric tissues by immunohistochemistry method (Figure 3 and Table 1). Moreover, we documented that miR-204 and SOX4 were negatively correlated in tissues from gastric cancer patients (Table 3). We for the first time made comprehensive analysis of miR-204 and SOX4 with clinicopathological parameters in patients and determined that miR-204 and SOX4 were dramatically associated with lymph node metastasis and tumor stages (Table 2).

The important roles of miRNAs played in tumorigenesis and metastasis become much clearer recent years. miRNAs contribute to nearly all kinds of human cancers including breast cancer, liver cancer, lung cancer, and gastric cancer [7–10]. In this study, we focused on miR-204 in gastric cancer. Sun et al. determined that miR-204 inhibits invasion and epithelial- mesenchymal transition in esophageal cancer [21]. Chung et al. documented that miR-204 mediates migration and invasion of endometrial cancer [39]. Mikhaylova et al. documented that miR-204 suppresses tumor growth in renal clear cell carcinoma [40]. Guo et al. documented that decreased expression of miR-204 in plasma is associated with a poor prognosis in patients with non-small-cell lung cancer [20]. In this study, we examined that miR-204 not only suppressed proliferation but also suppressed migration and invasion of gastric cancer cells. In other studies, Zhang et al., Zhang et al., and Liu et al. also documented that miR-204 was a suppressor in gastric cancer, which supported our findings [11, 22, 23]. Therefore, miR-204 played a comprehensive suppressing role in many kinds of human cancers. For further study of this miRNA, we made a systematical histological and pathological analysis of miR-204 in tissues from gastric cancer patients including comparing miR-204 level in normal tissues and gastric cancer tissues and analyzing the relationship between miR-204 level and clinicopathological parameters in gastric cancer patients. We for the first time determined that miR-204 was dramatically associated with lymph node metastasis and tumor stages. This is of great significance for clinical diagnosis and treatment.

SOX4 is one of the sex-determining region Y-box 4 (SOX4) genes and is a developmental transcription factor [24, 31, 41]. Numerous studies have found SOX4 plays an oncogenic role in many kinds of human cancers [24–29]. Bilir et al. demonstrated that SOX4 promoted tumor initiation and development in prostate cancer [25]. Song et al. documented that SOX4 was associated with malignant status of breast cancer [42]. Lin et al. showed that high expression of SOX4 was associated with poor outcome of colon cancer patients [26]. SOX4 was also demonstrated to promote gastric cancer in several studies [24, 29]. We also determined that SOX4 was overexpressed in human gastric cancer tissues compared with normal gastric tissues. And we for the first time document that SOX4 was associated with lymph node metastasis and tumor stages of gastric cancer.

As a target gene of miR-204, SOX4 was negatively correlated with miR-204 in tissues from gastric cancer patients. Previous studies showed that miR-204 suppressed gastric cancer through targeting USP47, RAB22A, SIRT1, Snai1, and so forth [11, 22, 23]. Getting together, in miR-204 regulation pathway, USP47, RAB22A, SIRT1, Snai1, and so forth and SOX4 may play an associating role contributing to gastric cancer progressing.

In a word, we documented miR-204 suppressed proliferation and metastasis of gastric cancer cells and found miR-204 and SOX4 were negatively correlated and associated with clinicopathological parameters in gastric cancer patients. These findings were helpful for better understanding the exact molecular pathways of miR-204 and SOX4 in gastric cancer and provided potential therapeutic targets for gastric cancer therapy.

## Figures and Tables

**Table 1:** Expression of miR-204 and SOX4 in gastric cancer and normal tissues.

Group	Positive expression		
	*n*	miR-204, *n* (%)	SOX4, *n* (%)
GC	54	19 (35.2)^*∗*^	39 (72.2)^*∗∗*^
Normal	54	34 (63.0)	17 (31.5)

Note: ^*∗*^*P* = 0.004; ^*∗∗*^*P* = 0.001.

**(a):** 

Parameter	*n*	miR-204 expression	
		Positive, *n* (%)	*P *value
Age (years)			
≤60	19	8 (42.1)	0.433
>60	35	11 (31.4)	
Gender			
Male	30	8 (26.7)	0.143
Female	24	11 (45.8)	
Tumor size (cm)			
≤5	32	13 (40.6)	0.313
>5	22	6 (27.3)	
Lymph node metastasis			
No	20	12 (60.0)	0.003
Yes	34	7 (20.6)	
Grade			
I	3	1 (33.3)	0.803
II	28	11 (39.3)	
III	23	7 (30.4)	
Stage			
I-II	19	10 (52.6)	0.048
III-IV	35	9 (25.7)	

**(b):** 

Parameter	*n*	SOX4 expression	
		Positive, *n* (%)	*P *value
Age (years)			
≤60	19	13 (68.4)	0.646
>60	35	26 (74.3)	
Gender			
Male	30	23 (76.7)	0.415
Female	24	16 (66.7)	
Tumor size (cm)			
≤5	32	22 (68.8)	0.492
>5	22	17 (77.3)	
Lymph node metastasis			
No	20	9 (45.0)	0.001
Yes	34	30 (88.2)	
Grade			
I	3	2 (66.7)	0.889
II	28	21 (75.0)	
III	23	16 (69.6)	
Stage			
I-II	19	9 (47.4)	0.003
III-IV	35	30 (85.7)	

**Table:** (a) Correlations (normal gastric tissues).

		miR-204	SOX4
miR-204	Pearson correlation	1	−.801^*∗∗*^
	Sig. (2-tailed)		.000
	*N*	54	54
SOX4	Pearson correlation	−.801^*∗∗*^	1
	Sig. (2-tailed)	.000	
	*N*	54	54

**Table:** (b) Correlations (gastric cancer tissues).

		miR-204	SOX4
miR-204	Pearson correlation	1	−.582^*∗∗*^
	Sig. (2-tailed)		.000
	*N*	54	54
SOX4	Pearson correlation	−.582^*∗∗*^	1
	Sig. (2-tailed)	.000	
	*N*	54	54

^*∗∗*^Correlation is significant at the 0.01 level (2-tailed).

## Abbreviations

miRNA: MicroRNA
mRNA: Messenger RNA
NC: Negative control
3′UTR: 3′ untranslated region.
